# Supplementary material for: Obscured inequity: How focusing on rates of disparities can conceal inequities in the reasons why adolescents are unvaccinated
Source: PLoS One. 2023 Nov 28;18(11):e0293928. doi: 10.1371/journal.pone.0293928 (PMC10684097; doi:10.1371/journal.pone.0293928)
Supplement: S1 Table — (DOCX) [file pone.0293928.s001.docx]

**Table S1: Sensitivity Analyses, Multinomial Logistic Regression Models Predicting Reason for Teen’s Unvaccinated Status for the HPV, MenACWY, and Tdap Samples, NIS-Teen 2012-2020.**

|  | **HPV** | **MenACWY** | **Tdap** |
| --- | --- | --- | --- |
| *Predicting Resource Failure (Agentic Refusal Base)* | OR  (CI) | OR  (CI) | OR  (CI) |
| Year | 0.81^***^ | 0.74^***^ | 0.73^***^ |
|  | (0.76, 0.87) | (0.69, 0.79) | (0.66, 0.82) |
| Year^2^ | 1.02^***^ | 1.02^***^ | 1.02^*^ |
|  | (1.01, 1.03) | (1.01, 1.02) | (1.00, 1.03) |
| Male | 1.91^***^ | 1.22^***^ | 1.14 |
|  | (1.68, 2.18) | (1.13, 1.31) | (0.98, 1.33) |
| Male × Year | 0.95 | -- | -- |
|  | (0.88, 1.02) |  |  |
| Male × Year × Year | 1.00 | -- | -- |
|  | (0.99, 1.01) |  |  |
| Race/ethnicity *(White reference)* |  |  |  |
| Hispanic | 1.32^***^ | 1.14 | 1.45^**^ |
|  | (1.20, 1.45) | (1.00, 1.30) | (1.15, 1.82) |
| Multiracial/other race | 1.45^***^ | 1.09 | 1.19 |
|  | (1.31, 1.60) | (0.96, 1.25) | (0.91, 1.56) |
| Black | 1.28^***^ | 0.90 | 0.99 |
|  | (1.16, 1.41) | (0.79, 1.03) | (0.78, 1.24) |
| Age | 0.99 | 0.95^***^ | 0.94 |
|  | (0.97, 1.01) | (0.93, 0.98) | (0.89, 1.00) |
| Family income *(> $75, 000 reference)* |  |  |  |
| Above poverty ≤ $75, 000 | 0.95 | 0.92 | 0.88 |
|  | (0.89, 1.01) | (0.85, 1.00) | (0.74, 1.05) |
| Below poverty | 1.25^***^ | 0.97 | 1.28 |
|  | (1.13, 1.38) | (0.85, 1.11) | (0.99, 1.65) |
| Mother’s education *(Less than high school reference)* |  |  |  |
| High school | 0.63^***^ | 0.77^**^ | 0.73^*^ |
|  | (0.55, 0.72) | (0.64, 0.93) | (0.55, 0.97) |
| Some college | 0.52^***^ | 0.70^***^ | 0.70^*^ |
|  | (0.45, 0.59) | (0.58, 0.83) | (0.52, 0.94) |
| College degree | 0.53^***^ | 0.77^**^ | 0.76 |
|  | (0.46, 0.60) | (0.64, 0.92) | (0.57, 1.03) |
| Census region *(Northeast reference)* |  |  |  |
| Midwest | 0.92^*^ | 0.99 | 0.92 |
|  | (0.85, 1.00) | (0.89, 1.10) | (0.74, 1.15) |
| South | 0.97 | 1.06 | 1.03 |
|  | (0.90, 1.04) | (0.96, 1.18) | (0.84, 1.27) |
| West | 0.96 | 1.01 | 0.81 |
|  | (0.87, 1.06) | (0.89, 1.16) | (0.62, 1.07) |
| Not vaccinated against…. *(missing only dependent variable vaccine)* |  |  |  |
| Tdap & dependent variable vaccine | 1.10 | 0.61^***^ | -- |
|  | (0.86, 1.40) | (0.51, 0.73) |  |
| MenACWY & dependent variable vaccine | 0.86^***^ | -- | 0.74 |
|  | (0.80, 0.93) |  | (0.54, 1.02) |
| HPV & dependent variable vaccine | -- | 0.46^***^ | 0.61^***^ |
|  |  | (0.41, 0.51) | (0.46, 0.80) |
| HPV, MenACWY & Tdap | 0.83^***^ | 0.32^***^ | 0.36^***^ |
|  | (0.76, 0.91) | (0.28, 0.37) | (0.28, 0.45) |
| State vaccine mandate | 1.02 | 0.95 | 0.93 |
|  | (0.78, 1.32) | (0.87, 1.03) | (0.76, 1.13) |
| *Predicting Other Reason (Agentic Refusal Base)* | **HPV** | **MenACWY** | **Tdap** |
| Year | 1.01 | 1.10^*^ | 1.23^**^ |
|  | (0.95, 1.08) | (1.01, 1.20) | (1.08, 1.40) |
| Year^2^ | 1.01 | 0.97^***^ | 0.97^***^ |
|  | (1.00, 1.01) | (0.96, 0.98) | (0.95, 0.98) |
| Male | 0.95 | 1.12^*^ | 1.32^**^ |
|  | (0.82, 1.11) | (1.02, 1.23) | (1.09, 1.59) |
| Male × Year | 1.08 | -- | -- |
|  | (0.99, 1.17) |  |  |
| Male × Year × Year | 0.99 | -- | -- |
|  | (0.98, 1.00) |  |  |
| Race/ethnicity *(White reference)* |  |  |  |
| Hispanic | 1.06 | 1.09 | 0.88 |
|  | (0.96, 1.18) | (0.93, 1.29) | (0.66, 1.17) |
| Multiracial/other race | 1.11^*^ | 0.90 | 0.79 |
|  | (1.00, 1.24) | (0.76, 1.07) | (0.58, 1.07) |
| Black | 1.09 | 0.88 | 0.81 |
|  | (0.99, 1.21) | (0.75, 1.04) | (0.61, 1.06) |
| Age | 0.97^**^ | 0.97 | 1.01 |
|  | (0.95, 0.99) | (0.94, 1.01) | (0.94, 1.08) |
| Family income *(> $75, 000 reference)* |  |  |  |
| Above poverty ≤ $75, 000 | 0.95 | 0.87^**^ | 0.87 |
|  | (0.89, 1.02) | (0.79, 0.97) | (0.70, 1.07) |
| Below poverty | 0.97 | 0.76^**^ | 1.09 |
|  | (0.87, 1.08) | (0.63, 0.90) | (0.81, 1.47) |
| Mother’s education *(Less than high school reference)* |  |  |  |
| High school | 0.77^**^ | 0.95 | 0.66^*^ |
|  | (0.66, 0.90) | (0.75, 1.21) | (0.45, 0.97) |
| Some college | 0.75^***^ | 0.99 | 0.96 |
|  | (0.65, 0.88) | (0.79, 1.24) | (0.66, 1.39) |
| College degree | 0.85^*^ | 1.13 | 1.26 |
|  | (0.73, 0.99) | (0.90, 1.43) | (0.88, 1.82) |
| Census region *(Northeast reference)* |  |  |  |
| Midwest | 0.91^*^ | 1.06 | 1.10 |
|  | (0.84, 0.99) | (0.93, 1.21) | (0.85, 1.42) |
| South | 0.94 | 1.22^**^ | 1.07 |
|  | (0.87, 1.01) | (1.08, 1.39) | (0.84, 1.38) |
| West | 0.97 | 0.95 | 0.94 |
|  | (0.87, 1.08) | (0.81, 1.12) | (0.68, 1.30) |
| Not vaccinated against…. *(missing only dependent variable vaccine)* |  |  |  |
| Tdap & dependent variable vaccine | 0.90 | 0.63^***^ | -- |
|  | (0.70, 1.14) | (0.51, 0.78) |  |
| MenACWY & dependent variable vaccine | 0.82^***^ | -- | 8.70^***^ |
|  | (0.76, 0.89) |  | (5.85, 12.93) |
| HPV & dependent variable vaccine | -- | 0.68^***^ | 0.98 |
|  |  | (0.60, 0.77) | (0.63, 1.54) |
| HPV, MenACWY & Tdap | 0.82^***^ | 0.44^***^ | 2.85^***^ |
|  | (0.74, 0.90) | (0.37, 0.52) | (1.97, 4.13) |
| State vaccine mandate | 1.11 | 1.15^**^ | 1.15 |
|  | (0.86, 1.44) | (1.04, 1.28) | (0.90, 1.48) |
| *N* | 87,268 | 54,769 | 10,956 |

The results in Table S1 reassigns the “need more information” reason to the agentic refusal category. The odds ratios presented Table S1 and the odds ratios presented in Table S2 are substantively equivalent.

^a^Exponentiated coefficients.

^b^95% confidence intervals in parenthesis
