# Supplementary material for: Obscured inequity: How focusing on rates of disparities can conceal inequities in the reasons why adolescents are unvaccinated
Source: PLoS One. 2023 Nov 28;18(11):e0293928. doi: 10.1371/journal.pone.0293928 (PMC10684097; doi:10.1371/journal.pone.0293928)
Supplement: S2 Table — (DOCX) [file pone.0293928.s002.docx]

**S2 Table**

Table S2: Average Marginal Effects for the Multinomial Logistic Regressions Predicting Other Reason for Teen’s Unvaccinated Status for the HPV, MenACWY, and Tdap Samples, NIS-Teen 2012-2020.

|  | **HPV AME**  **(95% CI)** | **MenACWY AME (95% CI)** | **Tdap AME**  **(95% CI)** |
| --- | --- | --- | --- |
| Year | 0.02 | 0.00 | 0.01 |
|  | (0.01, 0.02) | (0.00, 0.01) | (0.01, 0.02) |
| Male *vs. female/other gender* | -0.02 | 0.00 | 0.03 |
|  | (-0.03, -0.01) | (-0.02, 0.01) | (0.01, 0.06) |
| Age | -0.01 | 0.00 | 0.01 |
|  | (-0.01, 0.00) | (0, 0.01) | (0, 0.01) |
| Teen’s race/ethnicity |  |  |  |
| Hispanic *vs. White* | -0.01 | -0.01 | 0.00 |
|  | (-0.03, 0.01) | (-0.02, 0.02) | (-0.02, 0.02) |
| Black *vs. White* | 0.00 | -0.01 | -0.03 |
|  | (-0.02, 0.01) | (-0.03, 0.01) | (-0.07, 0.00) |
| Multiracial/other race *vs. White* | -0.01 | -0.03 | -0.05 |
|  | (-0.03, 0.01) | (-0.05, -0.01) | (-0.09, -0.01) |
| Hispanic *vs. Black* | -0.01 | 0.01 | -0.02 |
|  | (-0.03, 0.01) | (-0.02, 0.03) | (-0.06, 0.03) |
| Hispanic *vs. other race* | 0.00 | 0.03 | 0.00 |
|  | (-0.02, 0.02) | (0.00, 0.05) | (-0.05, 0.05) |
| Black *vs. other race* | 0.01 | 0.02 | 0.02 |
|  | (-0.02, 0.03) | (-0.01, 0.04) | (-0.03, 0.06) |
| Family income^2^ |  |  |  |
| Below poverty *vs. > $75,000* | -0.02 | -0.04 | -0.01 |
|  | (-0.04, -0.01) | (-0.06, -0.02) | (-0.05, 0.03) |
| Below poverty *vs. above poverty ≤ $75,000* | -0.02 | -0.03 | 0.01 |
|  | (-0.04, 0.00) | (-0.05, -0.01) | (-0.03, 0.04) |
| > $75,000 vs. *above poverty ≤ $75,000* | 0.01 | 0.01 | 0.01 |
|  | (-0.01, 0.02) | (0.00, 0.03) | (-0.02, 0.04) |
| Mother’s education^2^ |  |  |  |
| High school *vs. less than high school* | -0.01 | 0.02 | -0.03 |
|  | (-0.03, 0.02) | (-0.01, 0.05) | (-0.08, 0.01) |
| Some college *vs. less than high school* | 0.01 | 0.04 | 0.02 |
|  | (-0.02, 0.03) | (0.01, 0.06) | (-0.03, 0.07) |
| College degree *vs. less than high school* | 0.03 | 0.05 | 0.06 |
|  | (0.00, 0.05) | (0.02, 0.07) | (0.01, 0.11) |
| Some college *vs. high school* | 0.01 | 0.02 | 0.05 |
|  | (0.00, 0.03) | (0.00, 0.03) | (0.02, 0.09) |
| College degree *vs. high school* | 0.03 | 0.03 | 0.09 |
|  | (0.02, 0.05) | (0.01, 0.04) | (0.06, 0.13) |
| College degree *vs. some college* | 0.02 | 0.01 | 0.04 |
|  | (0.01, 0.03) | (0.00, 0.03) | (0.00, 0.07) |
| Census region^2^ |  |  |  |
| Midwest *vs. Northeast* | -0.01 | 0.01 | 0.02 |
|  | (-0.02, 0.00) | (-0.01, 0.03) | (-0.01, 0.06) |
| South *vs. Northeast* | -0.01 | 0.03 | 0.01 |
|  | (-0.02, 0.00) | (0.01, 0.04) | (-0.03, 0.04) |
| West *vs. Northeast* | 0.00 | -0.01 | 0.01 |
|  | (-0.02, 0.02) | (-0.03, 0.01) | (-0.04, 0.05) |
| Not vaccinated against…^2^ |  |  |  |
| Tdap & dependent variable vaccine *vs. missing only dependent variable vaccine* | -0.03 | -0.01 | -- |
|  | (-0.07, 0.01) | (-0.04, 0.01) |  |
| MenACWY & dependent variable vaccine *vs. missing only dependent variable vaccine* | -0.03 | -- | 0.32 |
|  | (-0.04, -0.01) |  | (0.28, 0.36) |
| HPV & dependent variable vaccine *vs. missing only dependent variable vaccine* | -- | 0.03 | 0.02 |
|  |  | (0.02, 0.05) | (-0.01, 0.04) |
| HPV, MenACWY & Tdap *vs. missing only dependent variable vaccine* | -0.02 | 0.00 | 0.18 |
|  | (-0.04, -0.01) | (-0.02, 0.02) | (0.15, 0.2) |
| State vaccine mandate | 0.02 | 0.03 | 0.03 |
|  | (-0.03, 0.07) | (0.01, 0.04) | (-0.01, 0.07) |
| Predicted probability base | 0.26 | 0.20 | 0.21 |
| *N* | 87,163 | 54,726 | 10,947 |

^a^95% confidence intervals in parentheses.

^b^All comparisons not shown.
