# Supplementary material for: Obscured inequity: How focusing on rates of disparities can conceal inequities in the reasons why adolescents are unvaccinated
Source: PLoS One. 2023 Nov 28;18(11):e0293928. doi: 10.1371/journal.pone.0293928 (PMC10684097; doi:10.1371/journal.pone.0293928)
Supplement: S1 File — (DOCX) [file pone.0293928.s003.docx]

**S1 File**

**State vaccine mandate data**

This dataset represents state-level mandates for the HPV, MenACWY, and Tdap vaccines by school grade from 2012-2020. Because some states require the HPV vaccine for only female students, HPV mandates are constructed separately for male and females.

To construct this dataset, I started by using information from immunize.org (1–3). I used the following crosswalk between age and school grade, because the NIS-Teen only provided adolescent’s age, while school mandates were generally enforced by grade: 13 years old = 8^th^ grade; 14 years old = 9^th^ grade; 15 years old = 10^th^ grade; 16 years old = 11^th^ grade; and 17 years old = 12^th^ grade.

Vaccine mandates were almost always implemented at the beginning of the school year. However, because the NIS-Teen is collected from January-December of each year, I marked a state & grade as having a vaccine mandate only if the mandate was in place during the entire calendar year (e.g. if a state implemented a MenACWY vaccine mandate was implemented for 9^th^ graders in the 2016-2017 school year, the dataset would indicate that 9^th^ graders had a MenACWY mandate in 2017).

Most states implemented mandates for one grade, rather than requiring all students in all grades to receive the vaccine during the first year of the mandate. Consequently, when a state required a vaccine for all 6^th^ graders starting in September 2017, the dataset would indicate that 6^th^ graders were under a vaccine mandate in 2018. Then in 2019, the dataset would indicate that 6^th^ & 7^th^ graders were under vaccine mandate. This continues progressively over time.

The immunize.org websites indicate whether states have a mandate, who is covered by the mandate, and when the mandate was implemented. I validated each entry by searching for state Department of Health press releases and news articles to confirm the accuracy of each entry. There were multiple instances where information from immunize.org conflicted with external sources. In those instances, I used the date of implementation from the external sources (see Table for all instances).

**Table: Conflicts Between immunize.org and Externally Validated Vaccine Mandate Information.**

| Vaccine | State | Source |
| --- | --- | --- |
| HPV | District of Columbia | Department of Health. 2008. “Notice of Final Rulemaking.” *District of Columbia Register* 55(18). (https://immunization.doh.dc.gov/imm/2008_May_2_Final_Rulemaking_DC_Regs.pdf). |
| MenACWY | Indiana | Indiana State Department of Health. 2010. “School Immunization Requirements Quick Reference Guide.” (https://chirp.in.gov/chirp_files/docs/2010%20School%20quick%20reference%20chart-August%2031,%202010.pdf). |
| MenACWY | Iowa | Iowa Department of Public Health. 2017. “Meningococcal Secondary School Vaccine Requirement Questions and Answers.” (https://www.idph.iowa.gov/Portals/1/userfiles/39/Mening%20School%20Requirement%20Q%20%26%20A%201-27-17%20Final.pdf). |
| MenACWY | Kentucky | Madison County Health Department. 2011. “Updated Child Day Care and School Immunization Requirements.” (https://madisoncountyhealthdept.org/Documents/SchoolImmunizationRequirements.pdf). |
| MenACWY | Michigan | Associated Press. 2013. “Snyder Signs Legislation on Immunization Rules.” *The Oakland Press.* Retrieved August 17, 2022 (https://www.theoaklandpress.com/snyder-signs-legislation-on-immunization-rules/article_a38c3b4a-326e-5a6e-af7a-6ddeb9740d92.html). |
| MenACWY | New York | New York State Department of Health. 2016. “New Law Requires Meningococcal Vaccine for All New York Children Entering 7th and 12th Grades.” Retrieved August 17, 2022 (https://www.health.ny.gov/press/releases/2016/2016-08-31_new_meningococcal_vaccine_law.htm#:~:text=(August%2031%2C%202016)%20%2D,to%20a%20new%20state%20law.). |
| MenACWY | North Dakota | North Dakota Codes School Immunization Requirements Chapter 33-06-05-01 (2019). (https://ndlegis.gov/information/acdata/pdf/33-06-05.pdf). |
| MenACWY | Rhode Island | Rhode Island Codes Immunization and Testing for Communicable Diseases Chapter 14-000-018. (2009). (https://www.phasys.pitt.edu/SearchResults.aspx?type=solution&ArticleID=a6d79ebb-e709-433f-ada1-6f22686b03bb&displaycount=100000&displaystart=0&keyword=&operator=or&citation=CRIR%2014-000-018). |
| Tdap | Alaska | Holland, Clayton. 2009. “New Vaccination Requirements for 2009-2010 School Year.” Kenai Peninsula Borough School District. Retrieved August 17, 2022 (https://kpbsd.org/WorkArea/DownloadAsset.aspx?id=13928). |
| Tdap | California | County of Los Angeles Public Health. 2011. “Make Sure Your Tweens and Teens’ Vaccines Are Up-to-Date This Spring Break.”. Retrieved August 17, 2022 (http://publichealth.lacounty.gov/phcommon/public/media/mediapubdetail.cfm?unit=media&prog=media&ou=ph&prid=899&keywords=pertussis&row=25&start=1). |
| Tdap | Michigan | Michigan Codes School Code Chapter 380.1177. (2009). (http://www.legislature.mi.gov/(S(afusjw5stavp0qtn2vhzz0bu))/mileg.aspx?page=GetObject&objectname=mcl-380-1177). |
| Tdap | Minnesota | Minnesota Department of Health Immunization Program. 2013. “New Immunization Laws for Schools, Child Care, and Early Childhood Programs Begin September 2014.” (http://mnsaa.org/wp-content/uploads/2011/05/2014-Immunization-Laws.pdf). |
| Tdap | Ohio | Hodges, Richard. 2014. “In Re: Approved Means of Immunization Pursuant to Sections 3701.13 and 3313.671 of the Ohio Revised Code.” (https://cms7files1.revize.com/starkcountyoh/Document_center/school-health/Resources/Directors-Journal-School-Requirements.pdf). |
| Tdap | Virginia | American Academy of Pediatrics Virginia Chapter. 2010. “School & Day Care Minimum Immunization Requirements.” Immunizations. Retrieved August 17, 2022 (http://www.virginiapediatrics.org/immunizations/#:~:text=Effective%20July%201%2C%202006%2C%20a,to%2060%20months%20of%20age.).  Virginia Department of Health. 2018. “FAQ – Tdap School Vaccination Requirement.” (https://www.vdh.virginia.gov/content/uploads/sites/11/2019/05/EnglishTDAP_FAQ.pdf). |

**References**

1. Immunization Action Coalition. State Information: Tdap Vaccine Mandates for Elementary and Secondary Schools [Internet]. Immunize.org. 2020 [cited 2022 Sep 15]. Available from: https://www.immunize.org/laws/menin_sec.asp

2. Immunization Action Coalition. State Information: HPV Vaccine Mandates for Elementary and Secondary Schools [Internet]. Immunize.org. 2021 [cited 2022 Sep 15]. Available from: https://www.immunize.org/laws/menin_sec.asp

3. Immunization Action Coalition. State Information: MenACWY Vaccine Mandates for Elementary and Secondary Schools [Internet]. Immunize.org. 2021 [cited 2022 Sep 15]. Available from: https://www.immunize.org/laws/menin_sec.asp

Measure
