# Supplementary material for: Obscured inequity: How focusing on rates of disparities can conceal inequities in the reasons why adolescents are unvaccinated
Source: PLoS One. 2023 Nov 28;18(11):e0293928. doi: 10.1371/journal.pone.0293928 (PMC10684097; doi:10.1371/journal.pone.0293928)
Supplement: Attachment — Submitted filename: Obscured Inequity_review 1.pdf [file pone.0293928.s005.pdf]

Obscured Inequity: How Focusing on Rates of Disparities Can Conceal Inequities in the Reasons Why Adolescents Are Unvaccinated

Overall, this paper is well thought, thorough and important in addressing health disparities. I applaud the author for studying undervaccination and vaccine hesitancy together. This is very critical. The author has done an incredible job in clarifying terms. However, some definitions may need further explanation rather than broad classification. Not sure if this would be beyond the scope of the work, but it would be great to unpack "vaccine hesitancy/ refusal" spectrum since there is evidence that some vaccine hesitant parents may still vaccinate their children. Similarly, with "undervaccination", does the author mean 'incomplete dosage', 'untimely vaccinations', or both, given that the author uses data on unvaccinated/ partially vaccinated adolescents. This is an excellent paper that can be further strengthened by adding some information in the discussion section.

Introduction

1. Provide evidence for statement made in lines 44-47.

Background

2. Provide evidence for lines 73-74 or indicate it's connection with the prior paragraph
3. What framework does line 74 allude to?
4. Cite line 83-84; Lines 138-140
5. Consider rephrasing line 136 -138 so that it connects back to the need or hesitancy towards vaccination and connects to the prior sentence.

Methods

6. In Table 1- Other reasons – "*Intend to but have not yet*" could be considered vaccine hesitant based on some literature- Hence the need to specifically define/ explain your terms
7. What the difference between the 3rd bullet point in -other reasons- vs. the 1st bullet point on -Resource failure reasons?
8. The dependent and independent variables are not clear from the methods.
9. Clearly state the statistical analyses conducted in text, even though they are indicated in the tables.
10. Rephrase line 195-197 to describe why this type of analysis was chosen.
11. Include the chosen/ proposed confidence levels for the analysis conducted

Results

12. Include descriptive analysis in the methods section as well, since descriptive data is reported in the results section
13. Consider adding "unvaccinated adolescents" to the Table 2 table for clarity.
14. Were any of the findings statistically significant? This should be part of the results

Discussion

15. Your results section is good, but the discussion section should elaborate more on the findings. What do they tell us? Is it relevant for practice or policy or research? What other gaps should be addressed?
16. For example, with resource failure, what could be done to address this issue? What perspective do you have behind higher vaccination rates among those living below the poverty line? Is it related to Medicaid or insurance?
17. Consider exploring other studies to support your findings. How do your results compare with other studies? Some other studies to consider include
    https://www.ncbi.nlm.nih.gov/pmc/articles/PMC9026751/ by Ejezie et al
    https://www.ncbi.nlm.nih.gov/pmc/articles/PMC9026751/ by Walker et al
18. Probably inequities as well- Line 394
