# Supplementary material for: Obscured inequity: How focusing on rates of disparities can conceal inequities in the reasons why adolescents are unvaccinated
Source: PLoS One. 2023 Nov 28;18(11):e0293928. doi: 10.1371/journal.pone.0293928 (PMC10684097; doi:10.1371/journal.pone.0293928)
Supplement: Attachment — Submitted filename: Response to Reviewers.docx [file pone.0293928.s006.docx]

I would like to thank both reviewers for their thoughtful comments. All references to edited text lines refer to the “Revised Manuscript with Track Change” file.

| **Reviewer # 1** | Author’s Response |
| --- | --- |
| The article is of excellent quality. It is a very important topic for the scientific community. The small points I suggest improving are: | Thank you for your comments! |
| Present an international overview of the theme in the introduction (what was done similarly in other countries, or even what was not done); | I added a paragraph to address this on lines 55-65. |
| Improve the practical implications of the study (How important is the study for society? How important is the study for professionals? How important is the study for the health system? Etc..) | I revised the discussion section per this suggestion and those of reviewer 2. Lines 485-491 address your comments. |
| **Reviewer #2** |  |
| *Overarching comments* | |
| Overall, this paper is well thought, thorough and important in addressing health disparities. I applaud the author for studying undervaccination and vaccine hesitancy together. This is very critical. | Thank you very much! |
| The author has done an incredible job in clarifying terms. However, some definitions may need further explanation rather than broad classification. Not sure if this would be beyond the scope of the work, but it would be great to unpack “vaccine hesitancy/ refusal” spectrum since there is evidence that some vaccine hesitant parents may still vaccinate their children. Similarly, with “undervaccination”, does the author mean ‘incomplete dosage’, ‘untimely vaccinations’, or both, given that the author uses data on unvaccinated/ partially vaccinated adolescents. | I removed the “/refusal” term, because I agree that it could be confusing. I added text on lines 45-52 to clarify the definitions. |
| This is an excellent paper that can be further strengthened by adding some information in the discussion section. | Thank you! |
| *Introduction* | |
| 1. Provide evidence for statement made in lines 44-47 | I addressed this by clarifying the definitions with citations on lines 45-52. |
| *Background* | |
| 2. Provide evidence for lines 73-74 or indicate it’s connection with the prior paragraph | I clarified the connection with the prior paragraph (see line 92). |
| 3. What framework does line 74 allude to? | It refers to the “sociodemographic disparities” framework. I added this clarification on line 94. |
| 4. Cite line 83-84; Lines 138-140 | I addressed this, see lines 105 & lines 158-162. |
| 5. Consider rephrasing line 136 -138 so that it connects back to the need or hesitancy towards vaccination and connects to the prior sentence. | I addressed this, see lines 158-162. |
| *Methods* | |
| 6. In Table 1- Other reasons – “Intend to but have not yet” could be considered vaccine hesitant based on some literature- Hence the need to specifically define/ explain your terms | In addition to clarifying the definitions, I added text on lines 213-217 to clarify why this reason was classified as an “other reason.” |
| 7. What the difference between the 3rd bullet point in -other reasons- vs. the 1st bullet point on -Resource failure reasons? | These are the reasons provided by the CDC, with no additional context. I classified “Teen is not the appropriate age/provider indicated could vaccinate at older age,” because it is ambiguous as to whether it is a resource failure or agentic refusal reason. The first half of the reason could mean that the provider did not recommend the vaccine (which would be a resource failure reason), but the second half of the reason that the provider indicated that the teen could vaccinate at an older age has parallels to research on a way that providers recommend the HPV vaccine to in response to vaccine hesitant parents (see Fenton 2019, *Journal of Health and Social Behavior*). Because of this, I classified this in the “other” category. |
| 8. The dependent and independent variables are not clear from the methods. | I relabeled these sections in the Methods section |
| 9. Clearly state the statistical analyses conducted in text, even though they are indicated in the tables. | I clarified this on lines 271-273. |
| 10. Rephrase line 195-197 to describe why this type of analysis was chosen. | I clarified this on lines 224-230. |
| 11. Include the chosen/ proposed confidence levels for the analysis conducted | I included this on line 280. |
| Results | |
| 12. Include descriptive analysis in the methods section as well, since descriptive data is reported in the results section | I included this on line 271. |
| 13. Consider adding “unvaccinated adolescents” to the Table 2 table for clarity | I included this on line 307. |
| 14. Were any of the findings statistically significant? This should be part of the results | I discuss statistical significance p-values on line 281 and included stars for significance in Table 2. |
| *Discussion* | |
| 15. Your results section is good, but the discussion section should elaborate more on the findings. What do they tell us? Is it relevant for practice or policy or research? What other gaps should be addressed? | Thank you for this suggestion. I have significantly reorganized and expanded the discussion section.  I discuss the implications of my findings on other researchers on lines 449-459.  I also discuss what these findings mean for global vaccination efforts (see lines 486-490). |
| 16. For example, with resource failure, what could be done to address this issue? What perspective do you have behind higher vaccination rates among those living below the poverty line? Is it related to Medicaid or insurance? | I discuss research on the higher rates of vaccination initiation among those living below the poverty line on lines 482-484 and potential interventions on lines 490-492. |
| 17. 7. Consider exploring other studies to support your findings. How do your results compare with other studies? Some other studies to consider include https://www.ncbi.nlm.nih.gov/pmc/articles/PMC9026751/ by Ejezie et al https://www.ncbi.nlm.nih.gov/pmc/articles/PMC9026751/ by Walker et al | Thank you for this suggestion. I included the Ejezie et al. study that you mentioned. Unfortunately, the link to the second study mentioned directs me to the Ejezie et al. article. I added additional citations throughout the Discussion section – however, Zotero is not compatible with tracked changes. I list the additional citations included below. |
| 18. Probably inequities as well- Line 394 | I fixed this on line 435. |

**Newly Added References**

1. MacDonald NE. Vaccine hesitancy: Definition, scope and determinants. Vaccine. 2015 Aug 14;33(34):4161–4.
2. Yang YT, Delamater PL, Leslie TF, Mello MM. Sociodemographic predictors of vaccination exemptions on the basis of personal belief in California. Am J Public Health. 2016;106(1):172–7.
3. Smith PJ, Chu SY, Barker LE. Children who have received no vaccines: Who are they and where do they live? Pediatrics. 2004 Jul 1;114(1):187–95.
4. World Health Organization. World Health Organization News. 2019 [cited 2023 Oct 8]. Ten threats to global health in 2019. Available from: https://www.annualreviews.org/doi/10.1146/annurev-publhealth-090419-102240
5. World Health Organization. Global vaccine market report 2022: a shared understanding for equitable access to vaccines. Geneva, Switzerland; 2023.
6. Dong L, Nygård M, Hansen BT. Sociodemographic correlates of human papillomavirus vaccine uptake: Opportunistic and catch-up vaccination in Norway. Cancers. 2021 Jan;13(14):3483.
7. Budu E, Ahinkorah BO, Guets W, Ameyaw EK, Essuman MA, Yaya S. Socioeconomic and residence-based related inequality in childhood vaccination in Sub-Saharan Africa: Evidence from Benin. Health Sci Rep. 2023;6(4):e1198.
8. Drolet M, Deeks SL, Kliewer E, Musto G, Lambert P, Brisson M. Can high overall human papillomavirus vaccination coverage hide sociodemographic inequalities? An ecological analysis in Canada. Vaccine. 2016 Apr 7;34(16):1874–80.
9. Bedford H, Attwell K, Danchin M, Marshall H, Corben P, Leask J. Vaccine hesitancy, refusal and access barriers: The need for clarity in terminology. Vaccine. 2018 Oct 22;36(44):6556–8.
10. Ejezie CL, Osaghae I, Ayieko S, Cuccaro P. Adherence to the Recommended HPV Vaccine Dosing Schedule among Adolescents Aged 13 to 17 Years: Findings from the National Immunization Survey-Teen, 2019–2020. Vaccines. 2022 Apr 8;10(4):577.
